# *Plasmodium falciparum* population dynamics in East Africa and genomic surveillance along the Kenya-Uganda border

**DOI:** 10.1038/s41598-024-67623-4

**Published:** 2024-08-05

**Authors:** Ashley Osborne, Emilia Mańko, Harrison Waweru, Akira Kaneko, Kiyoshi Kita, Susana Campino, Jesse Gitaka, Taane G. Clark

**Affiliations:** 1 Department of Infection Biology, Faculty of Infectious and Tropical Diseases, London School of Hygiene & Tropical Medicine, London, UK; 2 School of Tropical Medicine and Global Health, Nagasaki University, Nagasaki, Japan; 3 Directorate of Research and Innovation, Mount Kenya University, Thika, Kenya; 4 Centre for Malaria Elimination, Mount Kenya University, Thika, Kenya; 5 Department of Parasitology, Graduate School of Medicine, Osaka Metropolitan University, Osaka, Japan; 6 Department of Microbiology, Tumor and Cell Biology, Karolinska Institutet, Stockholm, Sweden; 7 Faculty of Epidemiology and Population Health, London School of Hygiene & Tropical Medicine, London, UK

**Keywords:** Microbial genetics, Computational biology and bioinformatics

## Abstract

East African countries accounted for ~ 10% of all malaria prevalence worldwide in 2022, with an estimated 23.8 million cases and > 53,000 deaths. Despite recent increases in malaria incidence, high-resolution genome-wide analyses of *Plasmodium* parasite populations are sparse in Kenya, Tanzania, and Uganda. The Kenyan-Ugandan border region is a particular concern, with Uganda confirming the emergence and spread of artemisinin resistant *P. falciparum* parasites. To establish genomic surveillance along the Kenyan-Ugandan border and analyse *P. falciparum* population dynamics within East Africa, we generated whole-genome sequencing (WGS) data for 38 parasites from Bungoma, Western Kenya. These sequences were integrated into a genomic analysis of available East African isolate data (n = 599) and revealed parasite subpopulations with distinct genetic structure and diverse ancestral origins. Ancestral admixture analysis of these subpopulations alongside isolates from across Africa (n = 365) suggested potential independent ancestral populations from other major African populations. Within isolates from Western Kenya, the prevalence of biomarkers associated with chloroquine resistance (e.g. *Pfcrt* K76T) were significantly reduced compared to wider East African populations and a single isolate contained the *PfK13* V568I variant, potentially linked to reduced susceptibility to artemisinin. Overall, our work provides baseline WGS data and analysis for future malaria genomic surveillance in the region.

## Introduction

Despite decades of progress, and a drastic reduction in malaria burden worldwide since the 1990’s, malaria incidence rates in 2020 and 2021 had their most dramatic increase since the start of the millennium^1,2^. Increased incidence in 2020 led to an estimated 22 million additional malaria cases worldwide, resulting in a total of 254 million cases and 625,000 deaths, exceeding the previous years’ estimated deaths by 57,000. Although many malaria control programmes have been resumed following the end to strict COVID-19 pandemic precautions, these numbers have continued to remain elevated in 2021. East African countries bordering Lake Victoria, including Kenya, Tanzania, and Uganda, accounted for nearly 10% of all malaria cases worldwide in 2022, with an estimated 23.8 million cases and upwards of 53,000 deaths^1^. The *Plasmodium falciparum* parasite is estimated to be responsible for almost 100% of malaria cases within the region and is the leading cause of severe disease^3^.

Although malaria transmission and infection risk have significantly decreased in Kenya, low-elevation regions, particularly Western Kenya and the Lake Victoria basin, remain areas of intense transmission. Consequently, malaria continues to be a leading cause of death among children under 5 years of age in these regions^4,5^. This high malaria incidence is driven by a variety of environmental and geopolitical factors, including fluid human migration patterns across country borders for socio-economic reasons, as well as tropical weather and freshwater conditions that favour vector breeding^6,7^. These factors have complicated the implementation of targeted malaria control within the region. The border of Kenya and Uganda has become an area of particular concern in recent years as Uganda struggles with increases in malaria incidence and mortality, in addition to the confirmed emergence and local spread of clinically artemisinin resistant *P. falciparum* parasites^1,8^. Artemisinin resistance in Uganda jeopardises malaria control efforts across East Africa and highlights the need to perform regular large-scale surveys of parasite populations to monitor for the emergence or cross-border spread of antimalarial resistant parasites.

East African parasite populations have been well documented to form a genetic cluster when compared to parasite populations from other regions of Africa. In general, East African parasite populations also tend to exhibit strong genetic ancestral linkages with one another and transmission connectivity supported by their geographic proximity^5,9^. However, high resolution genome-wide parasite population analyses have been extremely limited in Kenya, Tanzania, and Uganda, with most genetic studies focusing on microsatellite markers to assess genetic diversity, and sparse whole genome sequencing (WGS) data available from Uganda^6,10–13^. Although appropriate for establishing baseline genetic diversity metrics, microsatellite markers are not able to provide high resolution insights into the dynamics of a population or across multiple populations^14^. Genome-wide variants, such as single nucleotide polymorphisms (SNPs), can be characterised from the WGS data, and provide fine-scale insights into population structure and loci under selection, which may be linked to drug resistance, host, and mosquito interactions.

To establish genomic surveillance along the Kenyan-Ugandan border and produce a high-resolution analysis of *P. falciparum* population dynamics occurring within East Africa, we generated WGS data for parasites from the Western Kenyan border county of Bungoma, as well as performed a comprehensive analysis of East African isolates through a comparison with publicly available sequences^15^. Levels of identity-by-descent for isolates from Bungoma were compared to those from the surrounding regions to detect potential clonal outbreaks that may be occurring in the highland epidemic zones of the county. Population structure and ancestral analyses were performed to identify geographical associations. Further, the prevalence of biomarkers for resistance to antimalarials, such as chloroquine (e.g., *Pfcrt*), sulphadoxine (*Pfdhps*), pyrimethamine (*Pfdhfr*), and artemisinin (*PfK13*), were compared across East Africa. Overall, our study provides an in-depth and high-resolution analysis of the genomic diversity and ancestral origins of *P. falciparum* populations from across East Africa. In addition, it provides a baseline analysis of parasites from the Kenya-Uganda border.

## Results

### SNPs, genome-wide population data and multi-clonality

To assess the population structure of East African *P. falciparum* populations, SNP variants were identified from the WGS data of 599 *P. falciparum* isolates. These isolates were collected from Bungoma county in Western Kenya (n = 30/38), multiple points around the Lake Victoria basin (n = 160), Kenya (n = 74), Tanzania (n = 323), and Uganda (n = 12) (Supplementary Table S1; Supplementary Table S2). A total of 710,552 high-quality SNPs were called from non-hypervariable regions of the *P. falciparum* genome. The *F*_*ws*_ metric, or mean inbreeding coefficient, was calculated for each East African subpopulation to determine the proportion of complex infections amongst isolates and assess their within-host population diversity or assumed risk of out-crossing/inbreeding. Monoclonal *P. falciparum* isolates exhibit “high” *F*_*ws*_ estimates ≥ 0.95. Samples from Western Kenya (n = 30) had a mean *F*_*ws*_ coefficient of 0.851, with only 6 isolates exhibiting “high” *F*_*ws*_ estimates (Supplementary Figure S1). Low mean *F*_*ws*_ estimates are generally associated with higher proportions of complex infections and a high degree of panmixis within the population, which is common in Kenyan *P. falciparum* isolates from high transmission regions. In general, subpopulations across East Africa had mean *F*_*ws*_ coefficients ranging 0.738 to 0.907, with the lowest value (0.738) observed for isolates along the Kenyan Lake Victoria mainland, where transmission is high and stable throughout much of the year.

### *P. falciparum* isolates from highland epidemic outbreaks form distinct transmission clusters

**Figure 1:**
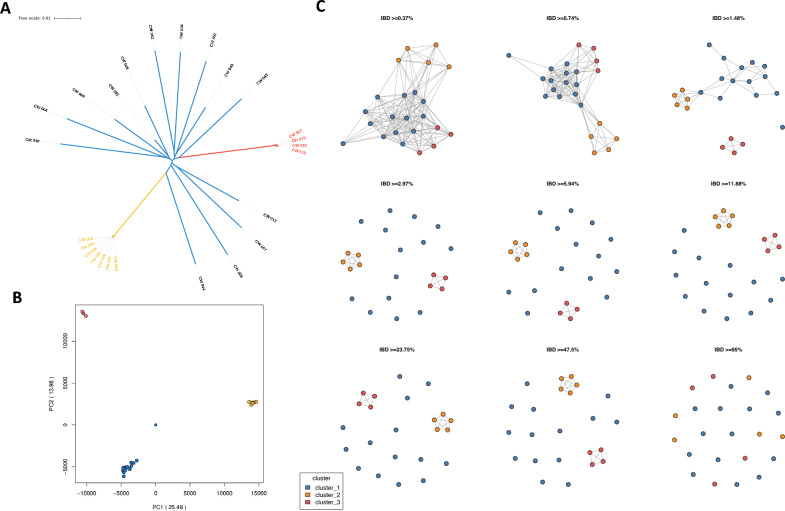
Population structure of *P. falciparum* isolates from highland epidemic outbreaks in Western Kenya. (**A**) Maximum likelihood tree of 30 isolates from Western Kenya (284,667 genome-wide SNPs). (**B**) PCA of the genetic pairwise matrix used to generate the maximum likelihood tree, with clusters coloured accordingly. (**C**) Pairwise identity-by-descent (IBD) connectivity plots between clusters, highlighting high levels of IBD (IBD > 47.5%) between clusters in Western Kenya.

Across the isolates of Bungoma county (n = 30), we identified 168,559 high-quality SNPs in non-hypervariable regions of the *P. falciparum* genome. A SNP-based principal component analysis (PCA) and maximum likelihood tree of these populations revealed one general population cluster consisting of 19 isolates (Cluster 1) and two distinct clusters comprising 7 isolates (Cluster 2) and 4 isolates (Cluster 3) (Fig. 1A, B). These observations suggest that the *P. falciparum* isolates in Clusters 2 and 3 may be highly genetically related or clones of each other.

Bungoma county spans both lake-endemic and highland epidemic zones, based on malaria endemicity classifications. Highland epidemic zones are prone to malaria outbreaks that can result in genetically similar *P. falciparum* transmission clusters^16^. To assess the relatedness of the 30 isolates, pairwise Identity-by-descent (IBD) was calculated to measure the proportion of pairs identical by descent at each SNP. Parasites sharing high proportions of long, unbroken segments of their genome, or demonstrating high levels of IBD, are generally classified as clones (IBD > 99%) or siblings (IBD > 47.5%). In contrast, more distantly related parasites share very few, shorter genome fragments^17,18^. Out of the 30 isolates, 25 passed quality thresholds required for IBD analysis. Parasites in the main population group (n = 14) demonstrated low levels of pairwise IBD (IBD < 2.97%), consistent with complex infections typically observed in high transmission regions. In contrast, isolates in Cluster 2 (n = 7) and Cluster 3 (n = 4) exhibited high IBD (IBD > 47.5%) with one another, classifying them as siblings (Fig. 1C)**.**

### Drug resistance

Non-synonymous mutations in genes associated with resistance to antimalarial drugs were analysed. The maximum *F*_*st*_ value for each SNP was calculated by comparing Western Kenyan isolates (n = 38) with other East African ones (n = 460) (Supplementary Table S3). Compared to the genome-wide analysis (n = 30), 3 to 8 additional isolates were potentially included in the analysis of candidate drug resistance loci, depending on whether variants were covered at least fivefold by sequence data. Variant frequencies from Western Kenyan isolates were also compared with those from the Kenyan region of Lake Victoria (n = 109) and West Africa (n = 50). A non-synonymous SNP on the *PfK13* gene resulting in the variant V568I was identified in 1 out of 37 Western Kenyan isolates. While the V568G variant has been identified as an in vitro candidate marker of reduced susceptibility to artemisinin, the impact of the V568I variant on artemisinin tolerance has not yet been characterised. An *in-silico* protein model of *Pfk13* was generated, including the wild-type position V568, the WHO candidate variant V568G, and the V568I variant observed within the Western Kenyan isolate. This analysis suggested that V568I could be a variant of concern (Supplementary Figure S2), but functional characterisation using experimental approaches is required to confirm this.

Resistance markers associated with chloroquine resistance were significantly reduced in Western Kenyan isolates (n = 38) compared to other East African isolates (n = 460). Specifically, only 1 out of 36 Western Kenyan isolates (2.8%) contained the primary *Pfcrt* biomarker for resistance, K76T, whereas 14.1% of East African isolates (65/460) contained this variant. Regarding *Pfmdr1,* the N86Y variant was absent in Western Kenyan isolates but had a minor allele frequency of 5.9% in East African isolates. It is hypothesised that the reference N86 is selected for by the use of lumefantrine and may reduce susceptibility to lumefantrine, piperaquine, and mefloquine^19^. Additionally, the Y184F and D1246Y variants were observed at frequencies of 54.5% (18/33) and 10.8% (4/37) in Western Kenyan isolates, respectively, compared to 37.7% (173/460) and 5% (23/460) in East African isolates. Although the Y184F variant is not significantly associated with reduced susceptibility to lumefantrine, it is believed to be genetically correlated with the acquisition of a drug-resistance phenotype^19^.

Variants associated with resistance to sulphadoxine and pyrimethamine on *Pfdhps* and *Pfdhfr*, respectively, were identified at high frequencies, consistent with other East African populations^20^. Variants N51I, C59R, and S108N on *Pfdhfr* were observed in 100% of isolates from Western Kenya, while the I164L variant was identified in only 1 out of 38 isolates (2.2%). Variants on *Pfdhps* were also observed at high frequencies within Western Kenyan isolates, with S436H, G437A, and K540E occurring in 21.6%, 100%, and 87.1% of isolates, respectively. Haplotype analysis was performed for variants on *Pfdhfr* (N51I, C59R, S108N, and I164L) and *Pfdhps* (S436H, G437A, K540E, and A581G) within parasites containing a read depth of at least fivefold at each position (n = 31). The wild-type haplotype, NCSISGKA, was not observed in any of the screened isolates. The quintuple mutant ***IRN***IS***AE***A accounted for 74.2% of isolates (23/31). The sextuple mutant ***IRN***I***HAE***A was observed in 7 isolates, while another sextuple mutant, ***IRNL***S***AE***A, was observed in a single isolate (1/31).

### East Africa has *P. falciparum* subpopulations with distinct genetic structure

**Figure 2:**
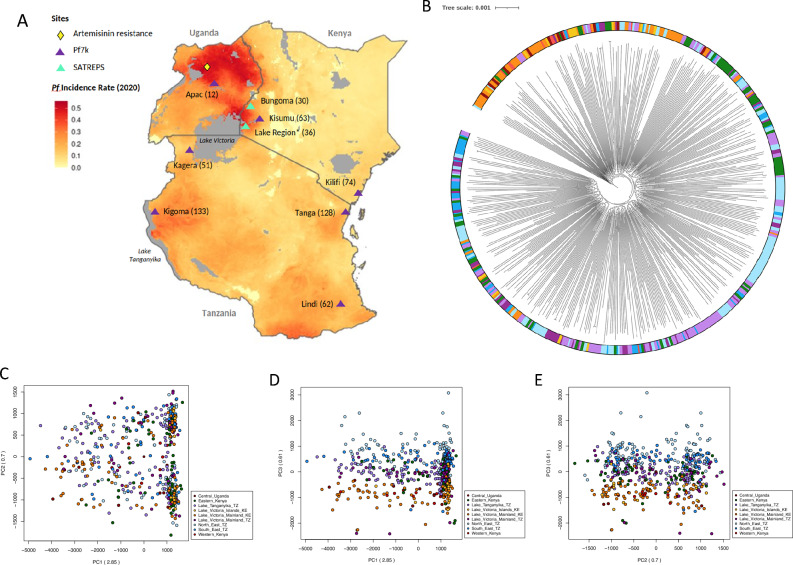
Genomic structure of *P. falciparum* isolates from East Africa form subpopulations. (**A**) Heatmap of *P. falciparum* incidence rates in 2020 across Kenya, Tanzania and Uganda with sampling sites and artemisinin resistance locations annotated (generated using *malariaAtlas* R-software). (**B**) A maximum likelihood tree for 587 isolates from Central Uganda, Eastern Kenya, Lake Victoria, Lake Tanganyika, North East Tanzania, South East Tanzania, and Western Kenya, based on 710,552 high-quality genome-wide SNPs. (**C**, **D**, and **E**) Principal component analysis (PCA) of East African subpopulations, showing the separation of isolates in PCs 1, 2, and 3.

A SNP-based maximum likelihood tree of 599 isolates from East Africa (Fig. 2A) revealed several subpopulations within the larger East African *P. falciparum* population (Fig. 2B). Isolates from the Lake Victoria region in Kenya (i.e., Kisumu, Mfangano island, Ngodhe island, and Suba district) (n = 109) clustered with isolates from Western Kenya (n = 30) and Uganda (n = 12), forming a distinct group separate from other East African subpopulations. Isolates from the Tanzanian region of Lake Victoria and Lake Tanganyika grouped more closely with isolates from North East Tanzania than with those from the Kenyan region of Lake Victoria. This population structure, identified by the maximum likelihood tree, was supported by a PCA, which also showed the separation of Kenyan Lake Victoria isolates, along with those from Western Kenya and Uganda, from other East African populations (Fig. 2C, D, E). There were no strong temporal trends to the clustering (Chi-squared P > 0.05), though it is important to note the limitations of using aggregated data in this context^15^.

### Ancestral admixture analysis reveals diverse ancestral origins of East African subpopulations

**Figure 3:**
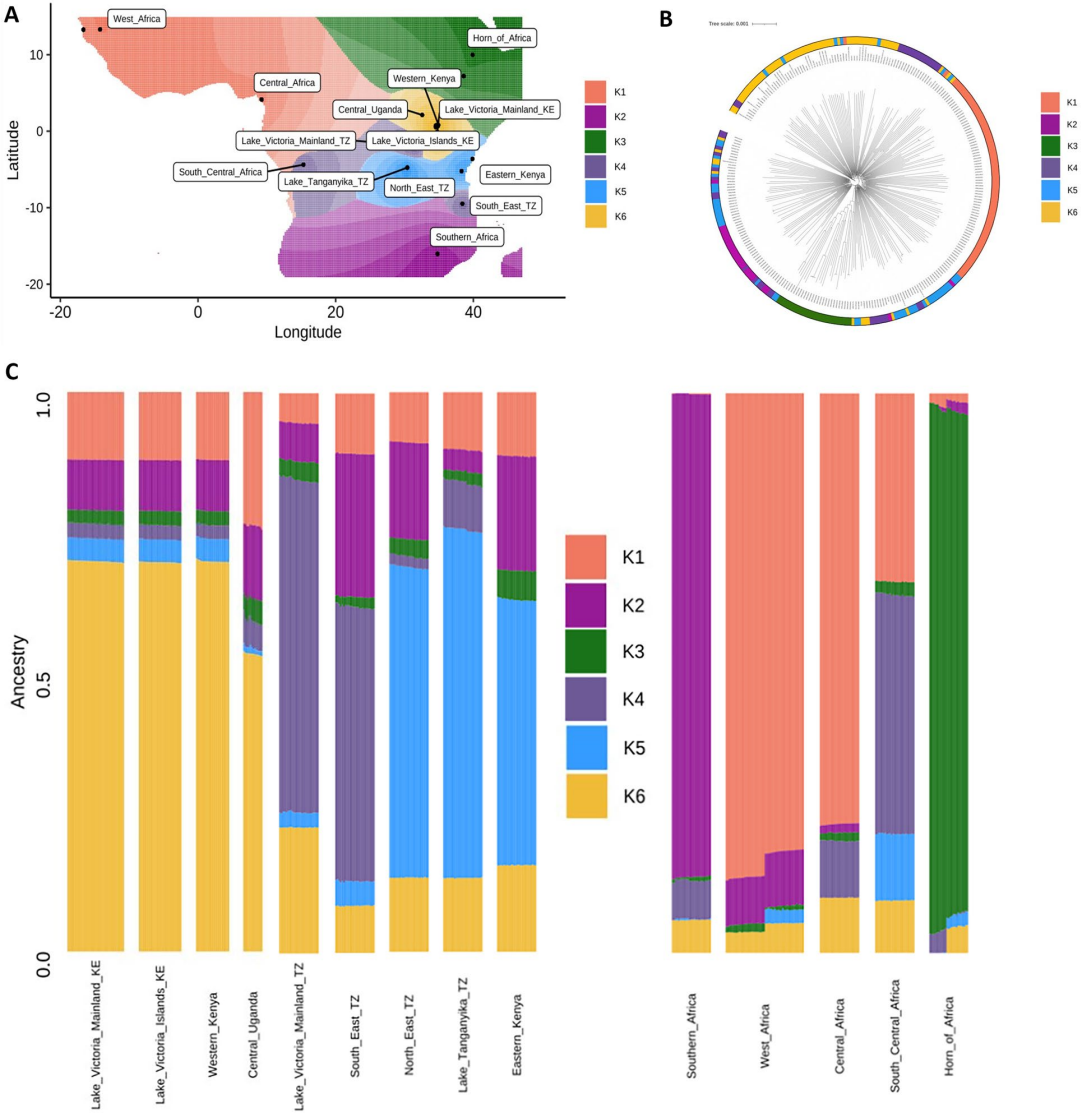
Genome-wide ancestral admixture analysis of East African *P. falciparum* subpopulations and regional parasite populations from across Africa. (**A**) Geographic map displaying ancestry coefficients, where *K* is estimated to represent 6 distinct ancestral populations across Africa. (**B**) Maximum likelihood tree of 363 isolates, based on 640,596 genome-wide SNPs, coloured according to their predominant K proportion. (**C**) Barplot showing ancestry proportions for each isolate (rows) within each subpopulation (columns).

**Figure 4:**
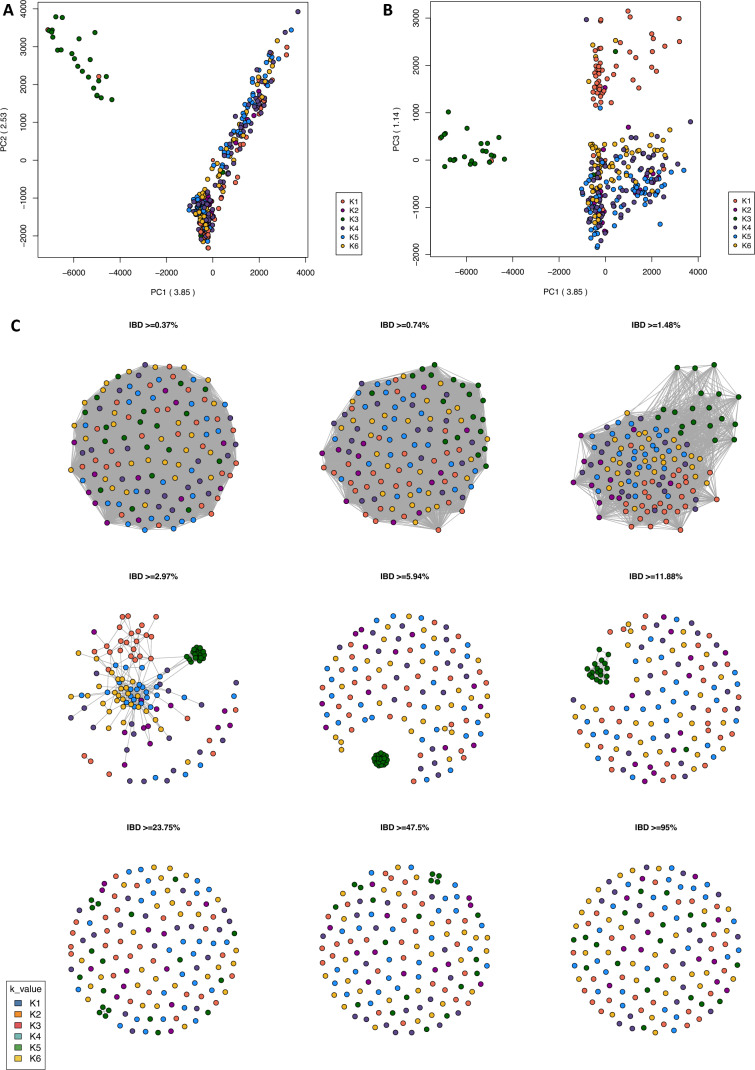
Connectivity between African *P. falciparum* ancestral populations. (**A**, **B**) Principal component analysis (PCA) generated based on pairwise genetic distance matrices of 640,596 high-quality genome-wide SNPs from 363 *P. falciparum* isolates. (**C**) Pairwise identity-by-descent (IBD) connectivity plots for isolates with an *F*_*ws*_ value > 0.90 (n = 293).

To evaluate the ancestral origins of East African *P. falciparum* subpopulations, genome-wide SNPs from isolates collected across the African continent (640,596 SNPs; n = 365 isolates) were analysed to infer ancestral genotype frequencies. These frequencies were combined with geographical coordinates to produce spatial models of allele sharing. Isolates were sourced from East Africa (Kenya, Tanzania, and Uganda; n = 218), West Africa (Guinea and The Gambia; n = 47), the Horn of Africa (Ethiopia; n = 25), Central Africa (Cameroon; n = 25), South Central Africa (Democratic Republic of Congo; n = 25), and Southern Africa (Malawi; n = 25) (Supplementary Table S2). With the optimum number of ancestral populations (K value) estimated to be 6 (K1–K6), the resulting admixture analysis revealed that isolates from East African subpopulations have distinct ancestral origins from one another, including two ancestral populations that appear independent from the wider African populations (Fig. 3). A maximum likelihood tree, PCA, and IBD connectivity plot were generated using the same genome-wide SNPs incorporated into the ancestral admixture analysis, supporting the identified population structure (Fig. 3B; Fig. 4; Supplementary Figure S3).

The K6 ancestral population exhibits high proportions of shared ancestry among isolates from Western Kenya (K6 proportion; 69.7%), Central Uganda (53.2%), and Lake Victoria isolates from Kenya (islands = 69.6%; mainland = 69.7%). In contrast, the K4 ancestral population, which is also observed in isolates from South Central Africa (e.g., DRC), differentiates Tanzanian Lake Victoria isolates (K4 proportion; 59.4%) from Kenyan Lake Victoria isolates (islands = 2.6%; mainland = 2.6%), Western Kenya (2.4%), and Uganda (3.0%) (Supplementary Figure S4). The K5 ancestral population links isolates from Eastern Kenya (proportion K5; 47.5%), North East Tanzania (55.5%), and Lake Tanganyika Tanzania (62.23%), distinguishing them from South East Tanzania (4.4%) and the Tanzanian Lake Victoria isolates (2.7%). The K2 ancestral population is prevalent in Southern Africa (86.4%) and is also present in South East Tanzanian isolates (25.6%) and Eastern Kenyan isolates (20.5%). South East Tanzanian isolates show a more mixed ancestry, with proportions of K1 (10.8%), K2 (25.6%), K3 (1.8%), K4 (4.4%), and K6 (8.4%). The Horn of Africa displays distinct ancestral origins from other African regions, with K3 representing the highest proportion of ancestry (91.6%).

### IBD analysis of ancestral populations reveals similar regions of homology across Africa

IBD statistics were calculated to characterise the structure of East African subpopulations at the chromosome level, by measuring the proportion of pairs identical by descent at each SNP across all isolates within the population. IBD was assessed according to the ancestral populations (K1–K6) identified through admixture analysis, due to the strong correlation between geographical proximity and genetic structure. As expected, isolates assigned to the K3 ancestral population (Horn of Africa) exhibited the highest fraction of pairwise IBD across the genome (mean = 0.08568, range = 0—0.85013), reflecting high genetic relatedness and genomic conservation among these isolates (Supplementary Table S4). In contrast, isolates from K6 (Western Kenya, Lake Victoria Kenya, and Central Uganda), K5 (Eastern Kenya, Lake Tanganyika Tanzania, and Lake Victoria Mainland Tanzania), and K4 (Lake Victoria Tanzania, South East Tanzania, and South Central Africa) displayed the lowest fractions of IBD, indicating lower genetic relatedness or reduced conservation of genomic regions (K6: mean = 0.00899, range = 0—0.12167; K5: mean = 0.01065, range = 0—0.09221; K4: mean = 0.00674, range = 0—0.01646). Genome-wide chromosome-level IBD values for ancestral populations (K1–K6) are presented (Supplementary Figure S5).

The top 5% of IBD positions for isolates from the K6 ancestral population were distributed across 16 regions on 4 chromosomes. For the K5 population, IBD positions were spread across 24 regions on 5 chromosomes, and for the K4 population, they were found in 7 positions on 3 chromosomes (Supplementary Table 5). Notably, within the K4 population, one of the IBD regions on chromosome 7 encompassed the *Pfcrt* gene, which is associated with partial resistance to chloroquine, a drug no longer prescribed as treatment for *P. falciparum*. In the K5 population, a region on chromosome 8 included the *Pfdhps* gene, which confers partial resistance to sulphadoxine-pyrimethamine (SP).

### Selection between *P. falciparum* subpopulations in East Africa

To identify variants under positive directional selection between subpopulations within East Africa, we analysed the genome-wide haplotype structure of isolates to pinpoint regions of high local homozygosity relative to neutral expectations. The integrated haplotype score (*iHS*) test statistic was used to detect regions with high local homozygosity, indicating positive selection within a single population (Supplementary Figure S6 A-C; Supplementary Table S6). Cross-population selection pressure was assessed using the *Rsb* metric, which compares extended haplotype homozygosity between populations (Supplementary Figure S6 D-F; Supplementary Table S7).

As commonly observed, SNPs under within-population positive selection in parasite populations were often in genes associated with host immune response and parasite immune evasion. Within K5 associated isolates (i.e. North East Tanzania, Lake Tanganyika, and Eastern Kenya), 5 genes of interest were identified with SNPs exhibiting significant *iHS* values ((− log10[1–2|ΦiHS–0.5|]) > 4.0). These included the heat shock protein 40 (HSP40), which is believed to play a role in parasite pathogenicity, and CX3CL1-binding protein 1, which is linked to the cytoadherence of infected erythrocytes (Supplementary Table S6). Cross-population selection analysis revealed common loci in comparisons across ancestral groups. Notably, a region on chromosome 6 containing a putative BFR1 domain-containing protein (*PF3D7_0617200*) and the AP-2 complex subunit alpha gene (*PF3D7_0617100*) showed significant differences between isolates from K1, K2, K4, K5 and K6 (number of SNPs: range 84–105; mean *Rsb* values: range 0.785–1.490) (Supplementary Table S7). Comparisons of K4 and K5 with K6 also identified positive selection in regions on the merozoite surface protein 3 encoding gene (*Pfmsp3*), known to be an important mediator of antibody responses and a candidate for malaria vaccines. Within the K4 population, 367 markers on the *Pfubp1* gene were identified with a mean *Rsb* of 0.419 when compared with K5, while 320 markers were identified within the K4 population when compared with K6, with a mean *Rsb* of 0.385.

## Discussion

Advances in next-generation sequencing platforms and the availability of large, publicly accessible datasets, has enabled high resolution insights into the genetic structure and ancestral origins of parasite populations within high transmission regions. Despite accounting for 10% of all malaria cases worldwide annually and the recent emergence of clinically artemisinin-resistant *P. falciparum* parasites in Uganda, East African parasite populations have been largely underrepresented in whole genome population studies^1,8^. To address this gap, we established genomic surveillance along the Kenyan-Ugandan border and supplemented the limited sequencing data available for Ugandan parasite populations by generating sequencing data for 38 *P. falciparum* isolates from Bungoma county in Western Kenya. This data was combined with newly available *P. falciparum* sequencing data from public repositories to produce a high-resolution analysis of the genetic structure and ancestral origins of parasite subpopulations in East Africa^15^. Our findings revealed decreasing frequencies of chloroquine resistance-associated biomarkers in Western Kenyan isolates compared to other East African subpopulations. Additionally, a non-synonymous variant on *pfk13* was identified within a codon flagged by the WHO as a candidate for artemisinin resistance^21^. Our analysis also uncovered subpopulations within East Africa with distinct genetic structures and diverse ancestral backgrounds from other major regional populations across Africa. While some temporal effects cannot be entirely ruled out, which can be challenging to assess and interpret using aggregated data^15^, the geographical signals in our findings are robust and significant.

Initial analysis of *P. falciparum* isolates from Bungoma county in Western Kenya identified revealed distinct genetic clusters within a region expected to have high genetic homogeneity^5^. A maximum likelihood tree using mapped sequence alignments identified two small clusters of samples from Bungoma that were distinct from other isolates from the county. These clusters appeared to be nearly genetically identical or clones of each other. Further investigation showed that Bungoma county is divided between two malaria endemicity classification zones: lake endemic and highland epidemic. Highland epidemic zones are prone to malaria outbreaks that can result in genetic clustering of *P. falciparum* clones within an area, while lake endemic zones sustain transmission throughout the year with peaks following the rainy seasons^4,16^. Estimated complexity of infection statistics reflected this and were broadly consistent with previous work showing that genetically mixed infections (lower *F*_*ws*_) are considerably more common in Africa than in other regions, related to the high intensity of malaria transmission in the continent^15^. Pairwise IBD analysis revealed that parasites in the main population group (n = 14) demonstrated low levels of pairwise IBD (IBD <  3.0%), consistent with infections typically observed in high transmission regions. In contrast, isolates in Cluster 2 (n = 7) and Cluster 3 (n = 4) exhibited high IBD (IBD > 47.5%) with one another, classifying them as siblings, and indicating an outbreak scenario. Further investigation of *P. falciparum* isolates from Bungoma county, along with higher resolution GPS data and integrated information on population movement, could provide greater insight into this phenomenon in future work. For our wider population analyses, one isolate from each cluster was included with isolates from the main Bungoma county population to prevent skewing based on the close genetic relatedness of the isolates.

Following the emergence of widespread resistance, chloroquine was discontinued as the first-line treatment for uncomplicated malaria^22^. A handful of countries that implemented swift and strict cessation of chloroquine usage, such as Malawi and Zambia, have documented the return of sensitive parasites. However, these results have not been universally observed^23–25^. The prevailing theory is that unauthorised over-the-counter sales of chloroquine may still be driving selection pressure for resistant parasites in regions where chloroquine is no longer the primary treatment method, particularly where *P. vivax* is not present. Isolates from Bungoma county in Western Kenya demonstrated a marked reduction in variants associated with chloroquine resistance, with only one isolate out of 36 containing the main K76T biomarker for resistance. This suggests the possibility that sensitive parasites may be returning to Kenya. Despite this reduction in chloroquine resistance biomarkers in Bungoma county, resistance-associated variants persist widely in Kenyan Lake Victoria isolates^5^. This persistence may be driven by continued sales of chloroquine across the lake basin, while its usage may be reduced in more isolated towns along the Kenya-Uganda border.

Although the presence of chloroquine resistance markers was reduced, variants associated with resistance to sulphadoxine-pyrimethamine (SP) were observed at high frequencies in Western Kenya, with many variants observed to be fixed within the parasite population. Haplotype analysis for variants on *Pfdhfr* (N51I, C59R, S108N, and I164L) and *Pfdhps* (S436H, G437A, K540E, and A581G) revealed no wild-type parasites (NCSISGKA) within the region. Instead, a quintuple mutant (***IRN***IS***AE***A) identified in approximately 75% of the population. The continued use of SP as an intermittent preventative treatment in pregnancy (IPTp) is likely driving this ongoing expansion and fixation of SP resistance within the population^1^. In addition to variants on *Pfdhps* and *Pfdhfr*, a non-synonymous variant on *Pfk13* was identified, resulting in a V to I amino acid substitution at codon 568. The V568G variant has been identified by the WHO as an in-vitro candidate marker of reduced susceptibility to artemisinin. However, the impact of the V568I variant on artemisinin tolerance has not yet been characterised^26^. An *in-silico* protein model of the wild-type position, WHO candidate, and V568I Western Kenya variant was generated to demonstrate structural variations. Despite these findings, in-vitro analysis of the V568I variant will be required to determine its impact, if any, on artemisinin susceptibility. Artemisinin resistance in Ugandan *P. falciparum* populations, coupled with the identification of this V568I mutant, underscores the need for regular monitoring of parasites within the region. This is crucial to preserve the efficacy of existing malaria treatment therapies.

Previous characterisations of the genomic diversity of East African *P. falciparum* parasites has revealed a largely homogenous population structure linked to transmission connectivity and geographic proximity, and high levels of human and vector migration^5,9^. However, analysis of an extended dataset, including *P. falciparum* isolates from new regions throughout East Africa, has revealed multiple parasite subgroups within the region. Isolates from Western Kenya, the Kenyan region of Lake Victoria, and Central Uganda exhibited a distinct genetic structure compared to other East African parasites and were not closely linked to isolates from the Tanzanian region of Lake Victoria. In contrast, isolates from the Tanzanian territory of Lake Victoria clustered more closely with those from South East Tanzania, likely due to their relative geographic proximity. The *P. falciparum* incidence rate and the proportion of complex infections (or within-host diversity) were higher in isolates from Central Uganda and the Kenyan region of Lake Victoria compared to Tanzanian Lake isolates. These differences may drive variations in parasite populations, despite high human migration throughout the lake basin. Isolates from Lake Tanganyika, North East Tanzania, and Eastern Kenya seemed more closely linked with one another but still displayed high levels of homogeneity with other Tanzanian parasite populations.

Admixture analysis revealed the diverse ancestral origins of East African parasites, including two distinct ancestral populations that provide insight into the observed East African subgroups. Isolates from Western Kenya, Central Uganda, and the Kenya region of Lake Victoria shared high proportions of the same, seemingly independent, ancestral genome with one another. They also exhibited similar cumulative proportions of ancestral genome fragments from other East African and African populations. This independent ancestry supports their distinct genetic structure compared to other East African parasite populations. This may be attributed to the migration of the Luo people into Western Kenya and Uganda from South Sudan, while the remainder of East Africa and the Lake Victoria basin was largely settled by Bantu peoples originating from Central Africa^27–29^. Interestingly, this ancestral population contributed ancestral genome segments not only to other East African populations but also to parasite populations from West Africa, Central Africa, South Central Africa, Southern Africa, and the Horn of Africa. Admixture analysis further identified large ancestral genome fragments shared amongst isolates from Eastern Kenya, Lake Tanganyika, and North East Tanzania.

The prevailing theory regarding the evolution of *P. falciparum* infections in humans posits a cross-species transmission event from Western Gorillas to humans in Central Africa approximately 10,000 years ago. This transmission subsequently spread across Africa via human migration routes^30,31^. In our analysis, Central African isolates (e.g., from Cameroon) were found to share high proportions of their ancestral genome with West African isolates, aligning with westward Bantu migrations from Central Africa. These isolates also donated ancestral genome fragments to all East African subpopulations^32^. West African isolates (e.g., from the Gambia and Guinea) shared a proportion of their genome with South Central African isolates (e.g., from the DRC), likely due to historical human migration linked to colonisation and slavery during the French and Belgian occupation of Guinea and the DRC, respectively^33^. Additionally, isolates from the Tanzanian region of Lake Victoria and South East Tanzania exhibited a substantial amount of South Central African ancestral admixture, highlighting the complex migratory and genetic interconnections within these regions.

Analysis of IBD was conducted to further investigate the structure and selection dynamics within East African parasite populations. As commonly observed in high transmission populations undergoing intense recombination, the fractions of pairwise IBD were generally low and uneven across genome, except for conserved *Plasmodium* proteins and a few segments including drug resistance-associated loci and genes involved parasite invasion, such as *PfPKAr*. A region on chromosome 7, which includes the chloroquine resistance transporter gene (*Pfcrt*), was identified in the top 5% of IBD for isolates from Lake Victoria in Tanzania, South East Tanzania, and South Central Africa. Although chloroquine has been discontinued for treating *P. falciparum* in this region, illegal use of the drug and potentially limited fitness costs for the parasite can contribute to the conservation of resistance in certain populations. Similarly, a region on chromosome 8 encompassing the gene *Pfdhps* was found in the top 5% of IBD for isolates from Eastern Kenya, North East Tanzania, and Lake Tanganyika (ancestral population K5). The high conservation of *Pfdhps* across East African parasite populations is likely driven by the continued use of SP for IPTp, supported by the high frequency of nearly fixed drug resistance-associated variants within the population. As expected, high IBD was observed across the genome for isolates from the Horn of Africa, reaffirming their high levels of genetic relatedness and distinct population structure compared to other sub-Saharan African parasite populations. This distinct population structure provides a valuable comparison for understanding the genetic diversity and evolutionary pressures within East African parasite populations.

To identify regions undergoing recent positive selection within and across East African parasite populations, we conducted a genome-wide analysis of haplotype structure, correlated with ancestral populations due to the strong relationship between geographical proximity and genetic structure. This analysis revealed variants under selection within genes largely associated with host immune response and parasite immune evasion. Notably, in isolates from Eastern Kenya, North East Tanzania, and Lake Tanganyika, genes in HSP40 and CX3CL1-binding proteins were identified. HSP40 is believed to play a role in determining parasite pathogenicity, while CX3CL1-binding protein 1 is linked to the cytoadherence of infected erythrocytes^34,35^. Cross-population analysis of positive selection highlighted a region under selection pressure with known associations to parasite immune evasion. Specifically, positive selection between the K4 and K5 ancestral populations, when compared to the K6 population, identified regions on the merozoite surface protein 3 encoding gene (*Pfmsp3*). This gene is a crucial  mediator of antibody responses and is associated with naturally acquired immunity, making it a popular candidate for malaria vaccines.

Overall, this study has offers an in-depth and high-resolution analysis of the genomic diversity and ancestral origins of *P. falciparum* populations from across East Africa, while also establishing a baseline of parasite populations from the Kenya-Uganda border. Despite certain limitations—such as limited data availability from Uganda, the use of retrospective samples from 2018, the convenience sampling nature of some collections, and genetic clustering of highland epidemic zone isolates from Bungoma county—we successfully quantify changes in drug resistance markers within parasite populations along the Kenya-Uganda border. Additionally, we identified distinct subpopulations within East Africa, each with its own unique genetic structure. This work not only provides a foundational set of sequence data but also serves as an exemplar for further genomic surveillance in the region and beyond.

## Materials and methods

### Study site selection

Dried blood spots were collected in 2018 from individuals residing in Bungoma county, Kenya (n = 53). Sample collection within this region was carried out as part of ongoing surveillance within the region, which includes regular community *P. falciparum* prevalence surveys. Bungoma county, located along the border with Uganda, features both lake-endemic and highland epidemic zones according to malaria endemicity classifications. Malaria prevalence within the lake-endemic zones is seasonally linked to short (October to December) and long (March to May) rainy seasons. Transmission in the highland epidemic regions results in malaria outbreaks, rather than continuous, seasonal transmission. Permission to conduct this study was obtained from the Mount Kenya University Independent Ethics and Research Committee (Approval reference: P609/10/2014). The study was performed in accordance with relevant guidelines and regulations. Educational workshops and sensitisation meetings were carried out within communities included in this study, prior to seeking community consent for study participation. Written informed consent was obtained from all study participants whose parasite DNA was used in this study.

### Study population characteristics

Bungoma county is occupied predominantly by Bukusu people, one of the Luhya Bantu tribes, who rely heavily on crop and livestock farming. The region’s primary economic crops are sugarcane and maize, while education and tourism sectors also employ a small proportion of the population. Bungoma county shares a border with Busia County, home to one of East Africa’s busiest border crossings, offering access to and from Rwanda, Burundi, the DRC, and South Sudan^36^.

### Species identification

Following WHO diagnostic guidelines, malaria species identification was carried out by microscopy at Mount Kenya University by trained microscopists and confirmed at the LSHTM using established nested PCR assays and in-house *Plasmodium* species prediction software^37^.

### Whole genome sequencing and bioinformatic processing

Out of the initial 53 isolates, *P. falciparum* DNA from 38 isolates (collected in 2018) was amplified using a selective whole genome amplification method with established protocols and primer sets. There were 15 samples with low-parasitaemia and poor DNA quality that disqualified them from successful WGS sequencing. After amplification, DNA was purified using KAPA Pure Beads and the associated DNA fragment clean-up protocol. Sequencing was performed on an Illumina NovaSeq 6000 platform by Eurofins Genomics in Germany, generating a minimum of 3.75 million paired reads per sample. The raw sequencing data was mapped to the PF3D7 *P. falciparum* reference genome (version 3) using *bwa-mem* software. Genomic variants were identified using *samtools* (version 1.12) and GATK (version 4.1.4.1) software suites, including *HaplotypeCaller* and *ValidateVariants*. Variants in low quality or low coverage regions, as well as highly variable regions, were discarded from analyses. For analysis of drug resistance SNPs, samples with less than fivefold read depth were not included. For genome-wide population analyses, samples with less than 70% mapping to the PF3D7 reference genome, or with less than a minimum coverage of fivefold coverage across 70% of the genome, were excluded. Of the 38 samples that were sequenced from Bungoma county, 30 were included in population-wide analyses with broader African *P. falciparum* populations, and between 33 and 38 isolates were assessed for drug resistance polymorphisms.

### Genomic variants and genome-wide population analyses

To assess genome-wide population variation, we generated pairwise genetic distance matrices for each population (East Africa, n = 599; Africa, n = 365) based on high-quality SNPs identified from isolates within each group. These matrices facilitated the creation of maximum likelihood trees using IQ-Tree (v2.1.4) and principal component analysis (PCA)^38^. The resulting maximum likelihood trees were visualised and annotated using the *iTOL* web-interface^39^. To identify functional variants associated with drug resistance, we employed *bcftools csq* (version 1.12), a haplotype-aware consequence caller, to annotate variants and infer their coding consequences^40^.

The R-based package *malariaAtlas* (version 1.5.1) was used to visualise *P. falciparum* incidence rates across Kenya, Tanzania, and Uganda^41^. Regional populations for cross-population analyses, including Western Kenya, Lake Victoria, West Africa, Horn of Africa, South Central Africa, Central Africa, and Southern Africa, were delineated based on previously characterised genetic clusters, geographic proximity, and documented patterns of human and vector migration^9^. In Bungoma county, isolates from outbreak Clusters 1 and 2 were found to be nearly identical. To avoid misleading population clustering due to close genetic distances, representative samples were taken from each cluster were selected and incorporated into region-wide population analyses.

To estimate ancestry proportions across regional populations in Africa (n = 365), continuous genetic variation was analysed using the R-based Tess3r package (version 1.1.0)^42–44^. Admixture calculations were performed by integrating genome-wide SNP data with geographical coordinates of the sequenced isolates. GPS coordinates for Bungoma isolates were recorded during collection, and for others are included in the publicly available Pf7 database metadata. Based on a cross-validation of 1 to 10 dimensions of eigenvalue decay, an optimum K value for ancestral admixture coefficients was six. Tess3r’s default parameters were employed, including a spatial regularisation parameter (σ = 1) to balance loss and penalty functions. The Tess3r software was run 50 times for K values ranging from 1 to 10, with the most accurate Q matrix of ancestry coefficients selected for each isolate. The analysis utilised the alternating projected least squares algorithm (APLS; method = “projected.Is”). Final plots were generated with the R-based package *ggplot2,* and spatial surfaces were interpolated using the R package *Krig* (version 15.2)^45–47^.

Complexity of infection, measured as the inbreeding coefficient metric (*F*_*ws*_), was evaluated using an in-house script designed to assess within-host diversity relative  to local population diversity. This metric estimates the fixation of alleles on a scale from 0 to 1, with an *F*_*ws*_ ≥ 0.95 indicating a clonal infection, while lower mean inbreeding coefficients suggest greater panmixia and higher complex of infections. The script calculates the likelihood of out-crossing or inbreeding, providing insights into the genetic diversity and structure of infections within individual hosts^48^.

To assess the connectivity and conservation of parasites from Bungoma county in relation to regional parasite populations, we calculated IBD fractions by measuring the pairwise shared ancestry of genomic segments. IBD fractions, represent segments of the genome that are inherited from recent common ancestors without intervening recombination events. Multi-allelic SNPs were excluded from IBD calculations. Recombination was accounted for using a hidden Markov model-based approach implemented in the *hmmIBD* software (version 2.0.0) to refine IBD estimates^49^. Recombination within the *P. falciparum* species is estimated to be 13.5 Kb per centiMorgan (cM), corresponding to chromosomal crossover events occurring at approximately an average rate of 1% per generation.

To identify regions of positive directional selection across the genome, we utilised the R-based *rehh* package (version 3.2.2). This package measures haplotype diversity metrics both within (*iHS*) or between (*Rsb*) populations. The *iHS* metric (integrated haplotype score) quantifies extended haplotype homozygosity at each SNP by comparing ancestral and derived alleles. A significant *iHS* value is indicated by a score > 4.0 ((− log10[1–2 | ΦiHS–0.5 |]) > 4.0). The *Rsb* metric evaluates pairwise population genetic distances, similar to the *F*_*st*_ metric but accounting for repeats in microsatellite alleles. A significant *Rsb* value is determined if -log10(p-value) > 5.

### Supplementary Information

Supplementary Information.

## Data Availability

Public accession numbers for raw sequence data analysed are contained in SRA studies ERP000190 and ERP000199, as well as accessible from the Pf3k project website (https://www.malariagen.net/apps/pf7/). Bungoma county raw sequences are available the ENA (project accession number TBC).
