# Supplementary material for: Plasmodium falciparum population dynamics in East Africa and genomic surveillance along the Kenya-Uganda border
Source: Sci Rep. 2024 Aug 5;14:18051. doi: 10.1038/s41598-024-67623-4 (PMC11300580; doi:10.1038/s41598-024-67623-4)
Supplement: Supplementary file 1 — Supplementary Information. [file 41598_2024_67623_MOESM1_ESM.pdf]

SUPPLEMENTARY FIGURES

Supplementary Figure S1. Multiplicity of infection across sampling sites included in the East Africa dataset (N = 599) calculated using the inbreeding coefficient (*F_{ws}* metric).

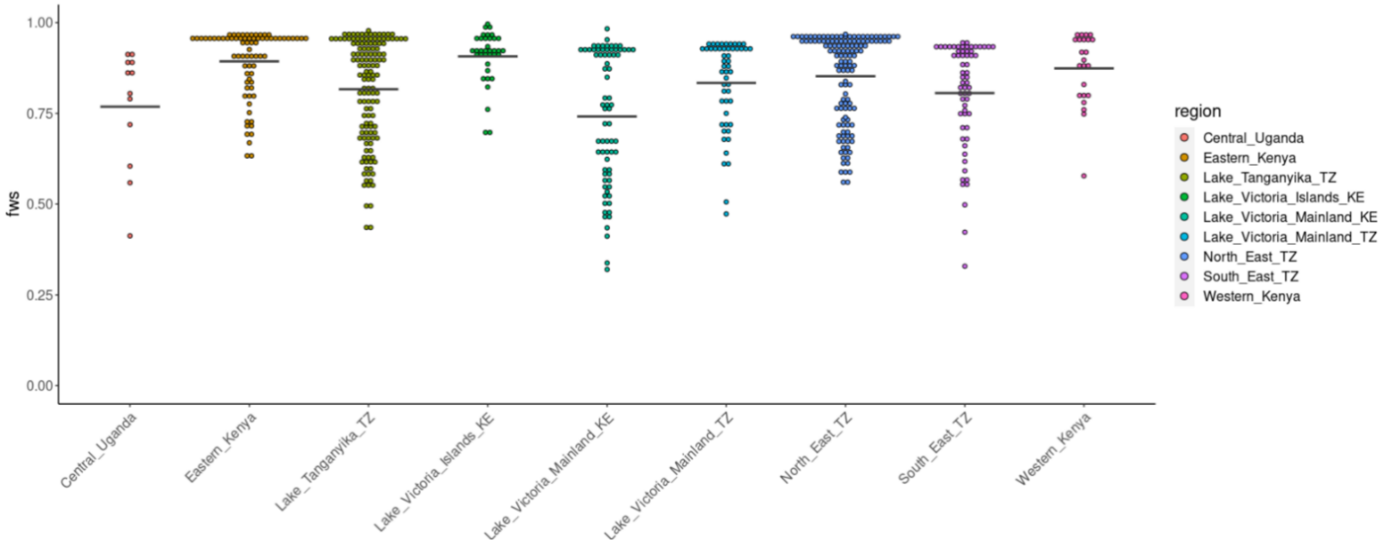

KE Kenya; TZ Tanzania

Supplementary Figure S2: An *in-silico* model of the protein structure of *P. falciparum* Kelch 13 (K13) protein highlighting codon 568, a WHO candidate for reduced susceptibility to artemisinin. (**A,D**) Wild-type codon Val568. (**B,E**) Codon 568 Gly [WHO candidate]. (**C,F**) Codon 568 Ile [identified in an isolate from Bungoma county, Kenya].

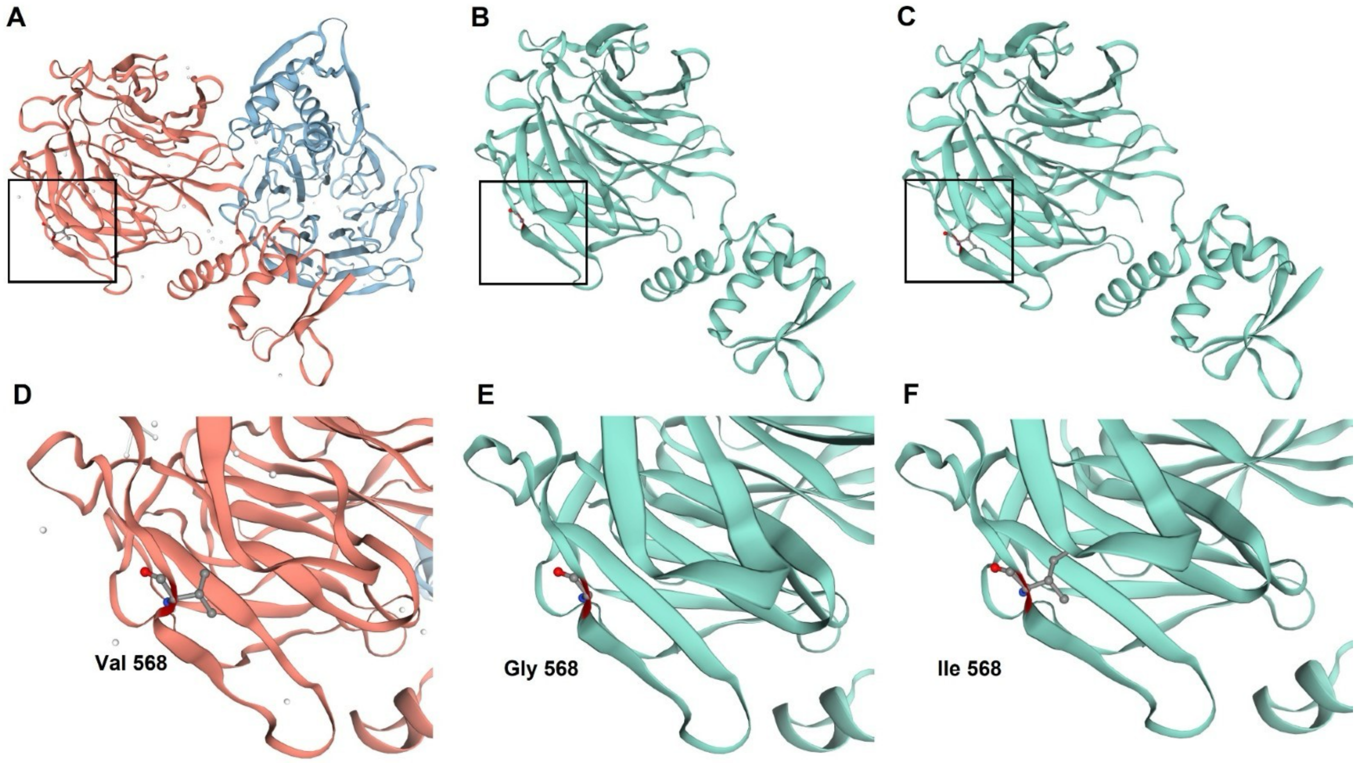

Supplementary Figure S3. Principal component analysis (PCA) of 365 *P. falciparum* isolates from across the African continent (640,596 genome-wide SNPs). (A and B) Isolates coloured by majority ancestral population (K1-K6). (C and D) Isolates coloured according to country of collection.

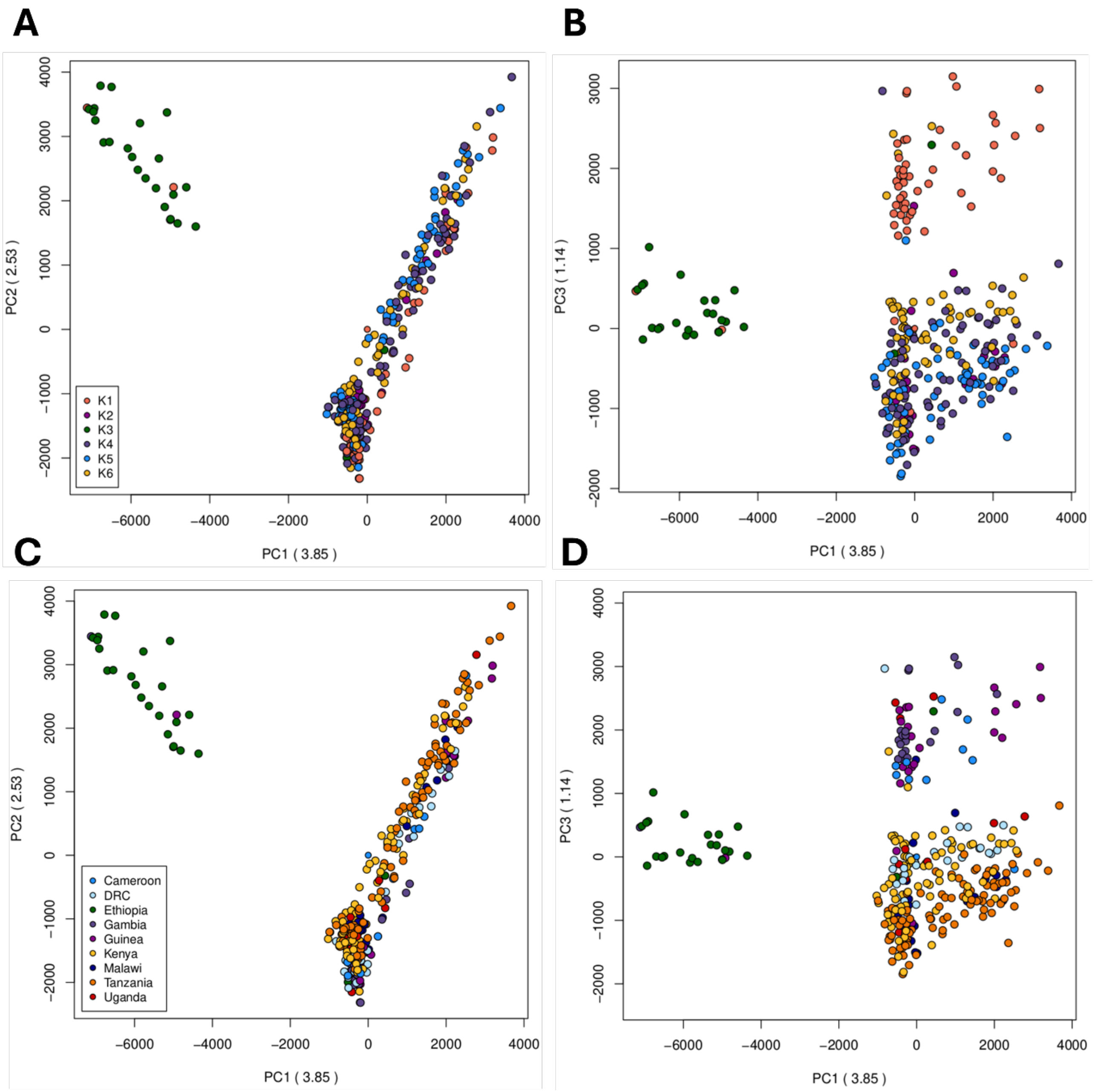

Supplementary Figure S4. Cumulative genome-wide ancestral admixture proportions for *P. falciparum* populations across Africa and subpopulations within East Africa. Cumulative percentages of ancestry where K is estimated to be 6.

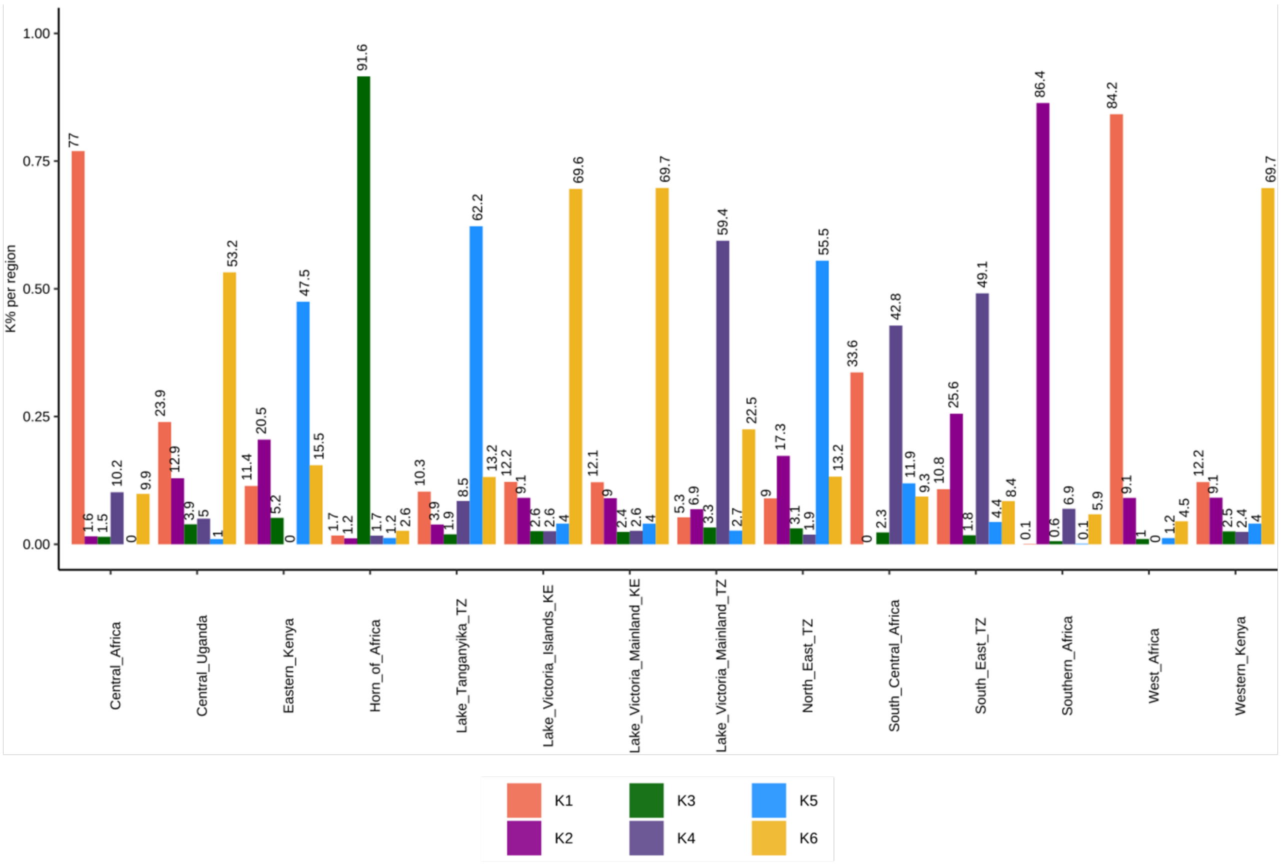

KE Kenya; TZ Tanzania

Supplementary Figure S5. Identity-by-descent (IBD) fractions in *P. falciparum* isolates from 6 estimated ancestral populations* within Africa. (**A**) IBD fractions across the genome by ancestral population. (**B**) IBD fractions along each chromosome by ancestral population.

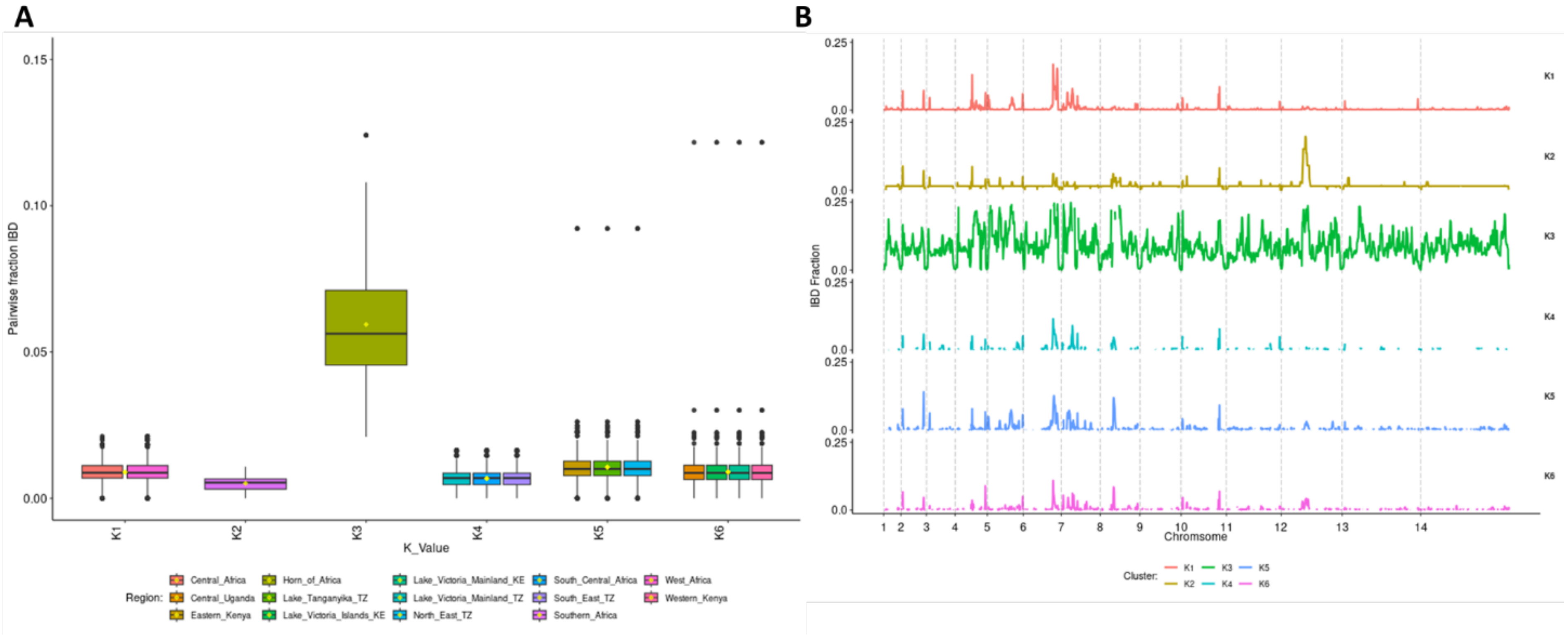

KE Kenya; TZ Tanzania; *K1 = Central Africa and West Africa; K2 = Southern Africa; K3 = Horn of Africa; K4 = South Central Africa, South East, TZ, Lake Victoria Mainland, KE, TZ; K5 = Eastern Kenya, Lake Tanganyika, TZ, North East, TZ; K6 = Central Uganda, Lake Victoria Islands, KE, Lake Victoria Mainland, KE, Western Kenya.

Supplementary Figure S6. Signatures of positive selection in East African subpopulations according to their maximum proportion ancestral population* (K-value). The genome-wide haplotype structure of isolates was analysed to locate regions of high local homozygosity relative to neutral expectation. (**A,B,C**) Variants undergoing within-population selection in the inferred K4, K5, and K6 populations with an *iHS* >4.0 ((-log10[1 – 2 | ΦiHS – 0.5 |]) > 4.0). (**D,E,F**) Cross-population analysis of variants under high positive directional selection (-log10(p-value) > 5).

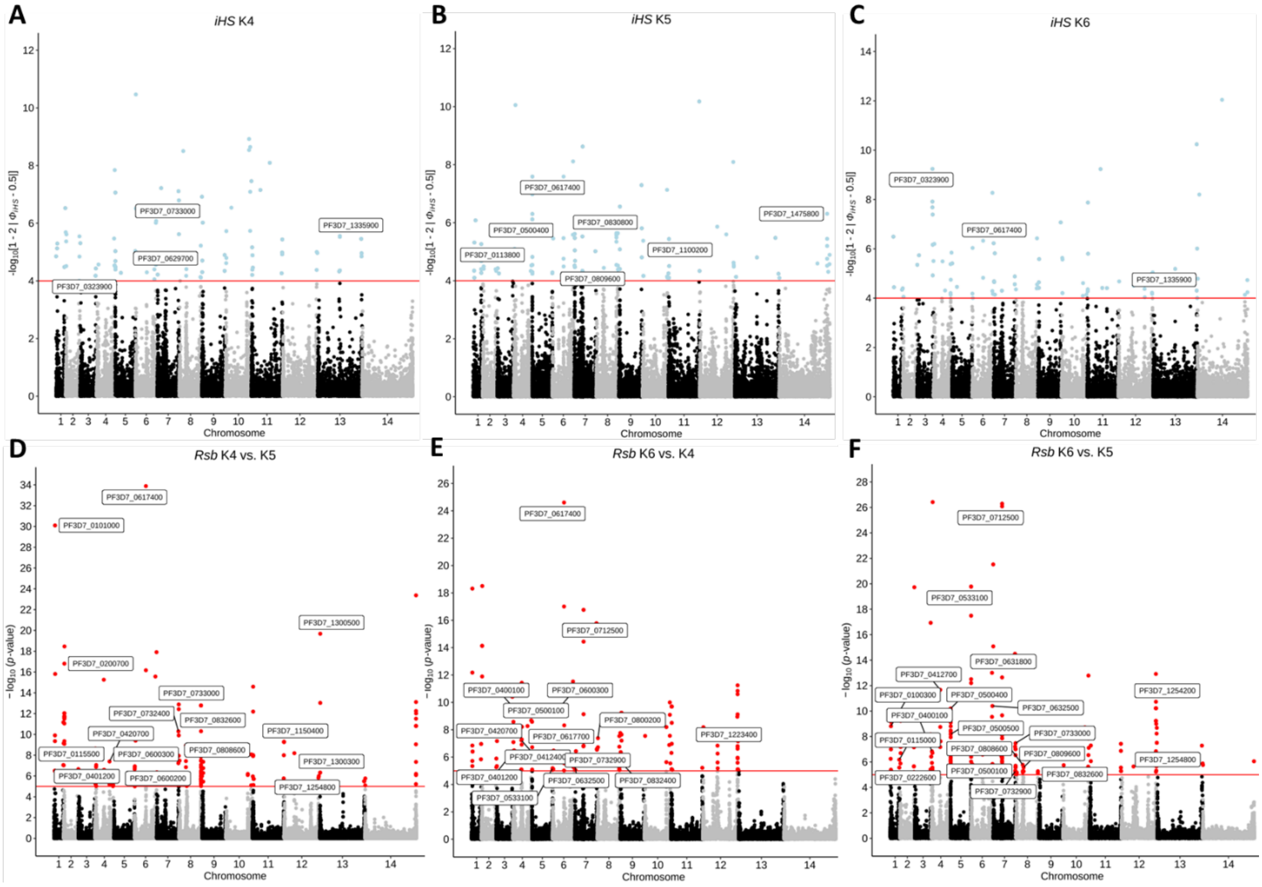

*K4 = South Central Africa, South East, TZ, Lake Victoria Mainland, KE, TZ; K5 = Eastern Kenya, Lake Tanganyika, TZ, North East, TZ; K6 = Central Uganda, Lake Victoria Islands, KE, Lake Victoria Mainland, KE, Western Kenya.

SUPPLEMENTARY TABLES

Supplementary Table S1. Summary data for 599 *P. falciparum* isolates included within East African genome-wide population analyses to characterise population dynamics and identify genetic markers of interest within isolates collected from Western Kenya.

Country	Site	Region	Year	Samples (N)
Kenya	Bungoma county	Western Kenya	2018	30
	Suba district	Lake Victoria mainland	2020	10
	Mfangano Island	Lake Victoria islands	2015, 2020	24
	Ngodhe Island	Lake Victoria islands	2016, 2020	12
	Kilifi	Eastern Kenya	2012	74
	Kisumu	Lake Victoria mainland	2014	63
Tanzania	Kagera	Lake Victoria mainland	2013	51
	Kigoma	Lake Tanganyika	2014	133
	Lindi	Southeast Tanzania	2013	62
	Tanga	Northeast Tanzania	2014	128
Uganda	Apac	Central Uganda	2010	12

Supplementary Table S2. Summary data for 365 *P. falciparum* isolates included within African genome-wide population analyses.

Region	Country	Year	Samples (N)
East Africa	Kenya (Western Kenya)	2018	21
	Kenya	2012, 2014, 2015, 2016, 2020	86
	Tanzania	2013, 2014	99
	Uganda	2010	12
West Africa	Guinea	2011	22
	Gambia	2014	25
Horn of Africa	Ethiopia	2013	25
South Central Africa	Democratic Republic of Congo	2014	25
Central Africa	Cameroon	2013	25
Southern Africa	Malawi	2011	25

Supplementary Table S3. Non-synonymous single nucleotide polymorphisms (SNPs) in known drug resistance genes. Known resistance-conferring variants highlighted in **bold.**

Gene	Position	Ref	Alt	Variant	Western Kenya N*	Western Kenya MAF	Max. F_{st}	East Africa MAF (*n* = 460)	LV Kenya MAF (*n* = 109)	West Africa MAF (*n* = 50)
Pfcrt	403291	G	T	D24Y	36	19.4	0.00327	7.7	7.3	11.0
	403625	A	C	**K76T**	36	2.8	0.00497	14.1	15.1	35.0
	403715	T	C	F106S	36	5.5	0	0	0.5	0
	404407	G	T	A220S	34	8.1	0.00139	14.5	15.1	35.7
	404836	C	G	**Q271E**	34	2.9	0.00770	14	14.8	35.7
	405362	A	G	N326S	34	0	0	0	0	0
	405600	T	C	I356T	34	0	0	0	0	22.0
	405838	G	T	**R371I**	34	2.9	0.00019	14.8	15.3	35.0
	406295	T	C	V418A	33	6.1	0.00005	0.1	1.0	0
Pfdhfr	748239	A	T	**N51I**	33	100	0.00244	95.1	99.1	96.0
	748262	T	C	**C59R**	33	100	0.00716	86.8	97.2	88.0
	748410	G	A	**S108N**	34	100	0.00021	99.6	99.1	100
	748577	A	T	**I164L**	38	2.2	0.00058	1.2	1.0	2.0
Pfdhps	549681	T	C	**S436H**	37	21.6	0.00006	0	0	0
	549685	G	C	**G437A**	37	100	0.00539	89.8	94.5	92.0
	549993	A	G	**K540E**	35	97.1	0.00337	89.7	95.0	49.0
	550117	C	G	**A581G**	37	0	0.00702	12.9	26.1	5.0
Pfmdr1	958145	A	T	**N86Y**	33	0	0.00297	5.9	3.3	17.0
	958440	A	T	**Y184F**	33	54.5	0.00006	37.7	44.4	48.0
	958484	A	T	T199S	32	3.1	0.01091	0.5	0.5	0
	960702	T	A	F938Y	34	2.9	0.00032	7.1	1.8	1.0
	961625	G	T	**D1246Y**	37	10.8	0.00020	5.0	4.1	5.0
PfK13	1725266	C	A	A578S	37	5.4	0.00037	0.1	0	0
	1725296	C	T	**V568I**	37	2.7	0.01009	0	0	0
	1725752	T	C	I416V	36	2.8	0.00046	0	0	0
	1725848	G	A	H384Y	36	2.8	0.00046	0	0	0
	1726015	T	A	Y328F	36	2.8	0.00046	0	0	0
	1726226	A	T	L258M	37	5.4	0.00036	0	0	0
	1726234	C	T	R255K	38	2.6	0.00220	1.5	1.0	0
	1726432	T	G	K189T	35	28.6	0.01286	16.7	20.2	13.0

LV Lake Victoria; MAF minor allele frequency; * number of isolates with depth of coverage > 5-fold (maximum n=38)

Supplementary Table S4. Fraction of pairwise identity by descent (IBD) across the genome.

Admixture (K value)	Region	Mean IBD	Range
K1	Central Africa<br>West Africa	0.01119	0 - 0.92449
K2	Southern Africa	0.01757	0 - 0.82675
K3	Horn of Africa	0.08568	0 - 0.85013
K4	South Central Africa<br>South East Tanzania<br>Lake Victoria Mainland, Tanzania	0.00674	0 - 0.01646
K5	Eastern Kenya<br>Lake Tanganyika, Tanzania<br>North East Tanzania	0.01065	0 - 0.09221
K6	Central Uganda<br>Lake Victoria Islands, Kenya<br>Lake Victoria Mainland, Kenya<br>Western Kenya	0.00899	0 - 0.12167

TZ Tanzania; KE Kenya

Supplementary Table S5. Top 5% of identity-by-descent (IBD) regions in East African *P. falciparum* populations by ancestral population*.

*K4 = South Central Africa, South East Tanzania, Lake Victoria Mainland (Kenya, Tanzania); K5 = Eastern Kenya, Lake Tanganyika Tanzania, North East Tanzania; K6 = Central Uganda, Lake Victoria Islands Kenya, Lake Victoria Mainland Kenya, Western Kenya.

Pop.	chr	start	end	Fraction	Gene Product	Gene Name
**K4**	6	1110001	1120000	0.11485	acetyl-CoA synthetase, putative	*ACS*
	6	1200001	1210000	0.05369	phospholipase, putative	*PL*
	7	380001	390000	0.04783	heat shock protein 86 family protein	-
	7	390001	400000	0.05848	heat shock protein 110	*HSP110c*
	7	390001	400000	0.05848	Cg8 protein	-
	7	400001	410000	0.06170	**chloroquine resistance transporter**	***CRT***
	7	420001	430000	0.07366	nucleic acid-binding protein, putative	-
**K5**	3	120001	130000	0.04329	cytoadherence linked asexual protein 3.2	*CLAG3.2*
	3	130001	140000	0.06240	cytoadherence linked asexual protein 3.1	*CLAG3.1*
	5	840001	850000	0.04293	single-stranded DNA-binding protein	-
	5	850001	860000	0.06154	adenosylhomocysteinase	*SAHH*
	5	870001	880000	0.06174	zinc finger protein, putative	-
	5	880001	890000	0.06582	ATP-dependent RNA helicase DDX1, putative	*DDX1*
	5	900001	910000	0.06886	transcription initiation factor TFIID subunit 10	*TAF10*
	6	1110001	1120000	0.06640	acetyl-CoA synthetase, putative	*ACS*
	6	1200001	1210000	0.06455	phospholipase, putative	*PL*
	6	1250001	1260000	0.05069	alpha/beta hydrolase, putative	-
	7	210001	220000	0.03587	HECT-type E3 ubiquitin ligase UT	*UT*
	7	220001	230000	0.05648	HECT-type E3 ubiquitin ligase UT	*UT*
	7	290001	300000	0.07217	importin-7, putative	-
	7	300001	310000	0.06765	conserved Plasmodium protein	-
	7	310001	320000	0.06154	conserved Plasmodium protein	-
	7	320001	330000	0.05766	conserved Plasmodium protein	-
	8	500001	510000	0.11938	JmjC domain-containing protein, putative	*JmjC1*
	8	510001	520000	0.11624	ribosomal protein L33, apicoplast, putative	-
	8	510001	520000	0.11624	JmjC domain-containing protein, putative	*JmjC1*
	8	540001	550000	0.07899	**hydroxymethyldihydropterin pyrophosphokinase-dihydropteroate synthase**	***PPPK-DHPS***
	8	540001	550000	0.07899	ATP-dependent RNA helicase DBP1, putative	*DBP1*
	8	550001	560000	0.04753	**hydroxymethyldihydropterin pyrophosphokinase-dihydropteroate synthase**	***PPPK-DHPS***
**K6**	6	1110001	1120000	0.10846	acetyl-CoA synthetase, putative	*ACS*
	7	420001	430000	0.05765	nucleic acid-binding protein, putative	-
	7	430001	440000	0.04455	prodrug activation and resistance esterase	*PARE*
	7	460001	470000	0.05460	conserved Plasmodium protein	-
	7	930001	940000	0.02909	40S ribosomal protein S5, putative	-
	8	500001	510000	0.08466	JmjC domain-containing protein, putative	*JmjC1*
	8	510001	520000	0.08183	ribosomal protein L33, apicoplast, putative	-
	8	510001	520000	0.08183	JmjC domain-containing protein, putative	*JmjC1*

	12	800001	810000	0.02890	cell cycle associated protein, putative	-
	12	900001	910000	0.03656	AP2 domain transcription factor AP2-G	*AP2-G*
	12	910001	920000	0.03968	AP2 domain transcription factor AP2-G	*AP2-G*
	12	920001	930000	0.04038	cAMP-dependent protein kinase regulatory sub.	*PKAr*
	12	990001	1000000	0.03706	histone chaperone ASF1, putative	*ASF1*
	12	990001	1000000	0.03706	polyadenylate-binding protein 1, putative	*PABP1*
	12	990001	1000000	0.03706	WD repeat-containing protein, putative	-
	12	990001	1000000	0.03706	WD repeat-containing protein, putative	-

Supplementary Table S6. SNPs on genes of interest under positive selection pressure within East African *P. falciparum* populations* (*iHS* value (-log10[1 – 2 | ΦiHS – 0.5 |]) > 4).

Pop.	chr	start	end	Markers (N)	Mean *iHS*	Gene ID (PF3D7_)	Gene products
**K4**	3	990000	1020000	51	0.907	0323800 (-); 0323600 (-)	conserved Plasmodium protein; BSD domain-containing protein, putative
	6	1220000	1250000	11	1.678	0629700 (SET1)	SET domain protein, putative
	6	1320000	1350000	84	0.803	0631900 (-)	stevor
	10	1600000	1630000	165	0.717	1040200 (-)	stevor
	13	1450000	1480000	50	0.797	1336200 (-)	conserved Plasmodium protein
**K5**	1	50000	90000	62	0.781	0100700 (-)	Plasmodium exported protein,
	1	520000	550000	202	0.731	0113700 (HSP40); 0114000 (EPF1); 0113900 (CBP1)	heat shock protein 40, type II; exported protein family 1; CX3CL1-binding protein 1
	6	710000	740000	67	1.278	0617200 (-); 0617600 (-); 0617100 (-)	BFR1 domain-containing protein, putative; stevor; AP-2 complex subunit alpha, putative
	6	1330000	1350000	144	0.832	0631900 (-)	stevor
	8	480000	510000	48	1.721	0809900 (JmjC1)	JmjC domain-containing protein, putative
	8	1300000	1330000	246	0.684	0830600 (-)	Plasmodium exported protein (PHISTc),
	10	1590000	1620000	186	0.672	1040200 (-)	stevor
	14	3110000	3140000	90	0.952	1475600 (BDP4)	bromodomain protein 4, putative
**K6**	3	990000	1030000	78	1.123	0323800 (-); 0323600 (-); 0324600 (-)	conserved Plasmodium protein; BSD domain-containing protein, putative; stevor
	6	710000	740000	31	1.347	0617200 (-); 0617600 (-); 0617100 (-)	BFR1 domain-containing protein, putative; stevor; AP-2 complex subunit alpha, putative
	6	1320000	1350000	105	0.735	0631900 (-)	stevor
	12	2200000	2240000	182	0.784	1254100 (-)	stevor
	13	1450000	1480000	59	0.660	1336200 (-)	conserved Plasmodium protein

Supplementary Table S7. Cross-population analysis of selection pressure between ancestral populations* (*Rsb* –log10 (p–value) > 5).

Pop.	chr	start	end	Markers (N)	Mean *Rsb*	Gene Name (PF3D7_)	Products
**K1|K2**	1	40000	70000	71	1.634	0100700 (-)	Plasmodium exported protein
**K1|K2**	3	110000	140000	186	0.776	0302500 (CLAG3.1); 0302200 (CLAG3.2)	cytoadherence linked asexual protein 3.1; cytoadherence linked asexual protein 3.2
**K1|K2**	4	1090000	1120000	180	1.064	0424300 (EBA165)	erythrocyte binding antigen-165
**K1|K2**	5	60000	90000	34	0.740	0501200 (PIESP2); 0501300 (SBP1)	erythrocyte surface protein; skeleton-binding protein 1
**K1|K2**	6	1270000	1300000	127	1.788	0631100 (-)	Plasmodium exported protein (PHISTb)
**K1|K2**	6	1320000	1350000	118	0.630	0631900 (-)	stevor
**K1|K2**	9	1460000	1490000	69	1.535	0937000 (-)	Plasmodium exported protein (PHISTb),
**K1|K2**	12	820000	850000	14	3.211	1221000 (SET10); 1220900 (HP1)	histone-lysine N-methyltransferase; heterochromatin protein 1
**K1|K2**	12	1000000	1030000	4	4.59	1225200 (-)	DNA-binding protein, putative
**K1|K3**	1	500000	530000	159	0.734	0113700 (HSP40)	heat shock protein 40, type II
**K1|K3**	2	870000	900000	66	0.738	0221800 (-)	hypothetical protein
**K1|K4**	1	20000	60000	137	1.514	0100700 (-)	Plasmodium exported protein
**K1|K4**	2	870000	900000	79	0.679	0221800 (-)	hypothetical protein
**K1|K4**	3	40000	70000	118	0.883	0300900 (-); 0300400 (-)	stevor; stevor
**K1|K4**	4	530000	600000	284	0.935	0412100 (mtRPS12)	ribosomal protein S12, mitochondrial
**K1|K4**	4	920000	950000	97	0.629	0420400 (RRF2); 0420300 (ApiAP2)	ribosome-recycling factor; AP2 domain transcription factor, putative
**K1|K4**	6	710000	740000	105	0.737	0617200 (-); 0617600 (-); 0617100 (-)	BFR1 domain-containing protein, putative; stevor; AP-2 complex subunit alpha, putative
**K1|K4**	6	1250000	1300000	173	2.414	0631100 (-); 0630100 (-)	Plasmodium exported protein (PHISTb); alpha/beta hydrolase, putative
**K1|K4**	6	1320000	1350000	164	0.788	0631900 (-)	stevor
**K1|K4**	10	1380000	1410000	376	0.642	1035400 (MSP3); 1034900 (MRScyt)	merozoite surface protein 3; methionine--tRNA ligase
**K1|K4**	10	1590000	1640000	357	0.931	1040200 (-)	stevor
**K1|K4**	12	1680000	1710000	123	1.075	1240000 (-)	3-hydroxyisobutyryl-CoA hydrolase
**K1|K5**	1	10000	80000	193	1.443	0100700 (-)	Plasmodium exported protein
**K1|K5**	6	1270000	1300000	133	2.400	0631100 (-)	Plasmodium exported protein (PHISTb)
**K1|K5**	6	1330000	1350000	187	0.739	0631900 (-)	stevor
**K1|K5**	8	490000	520000	36	3.192	0810100 (-); 0809900 (JmjC1)	ribosomal protein L33, apicoplast; JmjC domain-containing protein, putative
**K1|K5**	10	1380000	1410000	378	0.438	1035400 (MSP3); 1034900 (MRScyt)	merozoite surface protein 3; methionine--tRNA ligase
**K1|K5**	10	1590000	1640000	436	0.869	1040200 (-)	stevor
**K1|K5**	12	1680000	1740000	410	0.667	1240000 (-)	3-hydroxyisobutyryl-CoA hydrolase
**K1|K6**	6	710000	740000	84	0.796	0617200 (-); 0617600 (-); 0617100 (-)	BFR1 domain-containing protein, putative; stevor; AP-2 complex subunit alpha, putative
**K1|K6**	6	1250000	1300000	164	2.620	0631100 (-); 0630100 (-)	Plasmodium exported protein (PHISTb); alpha/beta hydrolase, putative
**K1|K6**	6	1320000	1370000	288	1.002	0631900 (-)	stevor
**K1|K6**	10	1380000	1410000	343	0.450	1035400 (MSP3); 1034900 (MRScyt)	merozoite surface protein 3; methionine--tRNA ligase
**K1|K6**	10	1590000	1620000	161	0.694	1040200 (-)	stevor

**K1|K6**	12	1680000	1710000	108	1.319	1240000 (-)	3-hydroxyisobutyryl-CoA hydrolase, put.
**K1|K6**	12	2200000	2230000	293	0.916	1254100 (-)	stevor
**K2|K4**	1	40000	80000	64	1.549	0100700 (-)	Plasmodium exported protein, unknown function, fragment
**K2|K4**	1	170000	200000	48	1.774	0104300 (UBP1); 0103900 (PIESP15)	ubiquitin carboxyl-terminal hydrolase 1; erythrocyte surface protein
**K2|K4**	3	110000	140000	169	0.707	0302500 (CLAG3.1); 0302200 (CLAG3.2)	cytoadherence linked asexual protein 3.1; cytoadherence linked asexual protein 3.2
**K2|K4**	4	1090000	1120000	179	1.024	0424300 (EBA165)	erythrocyte binding antigen-165, pseudo.
**K2|K4**	5	60000	90000	35	0.555	0501200 (PIESP2); 0501300 (SBP1)	erythrocyte surface protein; skeleton-binding protein 1
**K2|K4**	6	710000	740000	46	1.490	0617200 (-); 0617600 (-); 0617100 (-)	BFR1 domain-containing protein, putative; stevor; AP-2 complex subunit alpha, putative
**K2|K4**	6	1320000	1350000	88	0.875	0631900 (-)	stevor
**K2|K4**	9	1460000	1490000	72	1.472	0937000 (-)	Plasmodium exported protein (PHISTb)
**K2|K4**	10	1380000	1410000	270	0.807	1035400 (MSP3); 1034900 (MRScyt)	merozoite surface protein 3; methionine--tRNA ligase
**K2|K4**	12	900000	920000	9	3.900	1222600 (AP2-G)	AP2 domain transcription factor AP2-G
**K2|K4**	12	2190000	2240000	199	1.053	1254100 (-)	stevor
**K2|K5**	1	50000	80000	37	1.246	0100700 (-)	Plasmodium exported protein
**K2|K5**	1	170000	200000	41	2.210	0104300 (UBP1); 0103900 (PIESP15)	ubiquitin carboxyl-terminal hydrolase 1; erythrocyte surface protein
**K2|K5**	2	870000	900000	74	0.472	0221800 (-)	hypothetical protein
**K2|K5**	3	110000	140000	171	0.616	0302500 (CLAG3.1); 0302200 (CLAG3.2)	cytoadherence linked asexual protein 3.1; cytoadherence linked asexual protein 3.2
**K2|K5**	4	1090000	1120000	184	1.094	0424300 (EBA165)	erythrocyte binding antigen-165, pseudogene
**K2|K5**	5	60000	90000	38	0.599	0501200 (PIESP2); 0501300 (SBP1)	erythrocyte surface protein; skeleton-binding protein 1
**K2|K5**	9	1460000	1490000	64	1.500	0937000 (-)	Plasmodium exported protein (PHISTb)
**K2|K5**	10	1380000	1410000	281	0.854	1035400 (MSP3); 1034900 (MRScyt)	merozoite surface protein 3; methionine--tRNA ligase
**K2|K6**	1	30000	70000	61	1.808	0100700 (-)	Plasmodium exported protein
**K2|K6**	2	870000	900000	66	0.961	0221800 (-)	hypothetical protein
**K2|K6**	4	1090000	1120000	171	1.015	0424300 (EBA165)	erythrocyte binding antigen-165, pseudo.
**K2|K6**	6	1320000	1350000	86	1.092	0631900 (-)	stevor
**K2|K6**	9	1460000	1490000	65	1.406	0937000 (-)	Plasmodium exported protein (PHISTb)
**K2|K6**	10	1380000	1410000	255	0.658	1035400 (MSP3); 1034900 (MRScyt)	merozoite surface protein 3; methionine--tRNA ligase
**K3|K5**	1	500000	530000	144	0.637	0113700 (HSP40)	heat shock protein 40, type II
**K3|K5**	2	870000	900000	103	0.494	0221800 (-)	hypothetical protein
**K3|K6**	1	500000	550000	298	0.718	0113700 (HSP40); 0114000 (EPF1); 0113900 (CBP1)	heat shock protein 40, type II; exported protein family 1; CX3CL1-binding protein 1
**K3|K6**	2	870000	900000	95	0.703	0221800 (-)	hypothetical protein
**K4|K5**	1	50000	80000	82	1.936	0100700 (-)	Plasmodium exported protein
**K4|K5**	4	530000	560000	166	0.431	0412100 (mtRPS12)	ribosomal protein S12, mitochondrial
**K4|K5**	4	920000	950000	103	0.569	0420400 (RRF2); 0420300 (ApiAP2)	ribosome-recycling factor; AP2 domain transcription factor, putative
**K4|K5**	6	50000	70000	38	0.902	0601500 (-)	Plasmodium exported protein (PHISTb)
**K4|K5**	6	710000	740000	105	1.366	0617200 (-); 0617600 (-); 0617100 (-)	BFR1 domain-containing protein, putative; stevor; AP-2 complex subunit alpha, putative

**K4|K5**	6	1320000	1350000	157	0.570	0631900 (-)	stevor
**K4|K5**	8	480000	510000	60	1.999	0809900 (JmjC1)	JmjC domain-containing protein, putative
**K4|K5**	10	1380000	1410000	367	0.419	1035400 (MSP3);<br>1034900 (MRScyt)	merozoite surface protein 3;<br>methionine--tRNA ligase
**K4|K6**	1	30000	80000	119	1.585	0100700 (-)	Plasmodium exported protein
**K4|K6**	3	30000	60000	103	0.802	0300400 (-)	stevor
**K4|K6**	4	530000	590000	186	0.951	0412100 (mtRPS12)	ribosomal protein S12, mitochondrial
**K4|K6**	4	920000	950000	83	0.636	0420400 (RRF2);<br>0420300 (ApiAP2)	ribosome-recycling factor;<br>AP2 domain transcription factor, putative
**K4|K6**	6	710000	760000	88	1.403	0617900 (H3.3);<br>0617200 (-);<br>0617800 (H2A);<br>0617600 (-);<br>0617100 (-)	histone H3 variant;<br>BFR1 domain-containing protein, putative;<br>histone H2A;<br>stevor;<br>AP-2 complex subunit alpha, putative
**K4|K6**	10	1380000	1410000	320	0.385	1035400 (MSP3);<br>1034900 (MRScyt)	merozoite surface protein 3;<br>methionine--tRNA ligase
**K4|K6**	10	1600000	1630000	189	0.810	1040200 (-)	stevor
**K5|K6**	1	30000	70000	100	1.490	0100700 (-)	Plasmodium exported protein,
**K5|K6**	3	1000000	1030000	107	0.924	0323800 (-);<br>0324600 (-)	conserved Plasmodium protein<br>stevor
**K5|K6**	6	710000	740000	85	0.785	0617200 (-);<br>0617600 (-);<br>0617100 (-)	BFR1 domain-containing protein, putative;<br>stevor;<br>AP-2 complex subunit alpha, putative
**K5|K6**	6	1320000	1370000	267	0.994	0631900 (-)	stevor
**K5|K6**	8	480000	510000	55	1.538	0809900 (JmjC1)	JmjC domain-containing protein, putative
**K5|K6**	10	1600000	1650000	337	0.699	1040200 (-)	stevor
**K5|K6**	12	2190000	2280000	465	1.011	1254100 (-)	stevor
